# Effectiveness of proton pump inhibitor in unexplained chronic cough

**DOI:** 10.1371/journal.pone.0185397

**Published:** 2017-10-10

**Authors:** Hye Jung Park, Yoo Mi Park, Jie-Hyun Kim, Hye Sun Lee, Hyung Jung Kim, Chul Min Ahn, Min Kwang Byun

**Affiliations:** 1 Department of Internal Medicine, Gangnam Severance Hospital, Yonsei University College of Medicine, Seoul, Korea; 2 Biostatistics Collaboration Unit, Yonsei University College of Medicine, Seoul, Korea; University Hospital Llandough, UNITED KINGDOM

## Abstract

**Background:**

Current guidelines recommend that patients with unexplained chronic cough undergo empirical proton pump inhibitor (PPI) treatment, but scientific evidence for this treatment is lacking. We investigated the effectiveness and appropriate dose of PPI therapy in chronic cough.

**Methods:**

We included 27 patients with unexplained chronic cough after excluding subjects with positive response to postnasal drip medication. Subjects were randomized to a placebo, standard, and high dose of PPI groups with blinding. The drug or placebo was administered orally for 8 weeks, and the Leicester Cough Questionnaire (LCQ) score and visual analogue scale (VAS) scores were collected.

**Results:**

The LCQ score in the PPI group significantly improved from 0 weeks (11.4 ± 1.4) to 4 weeks (14.8 ± 1.4) and to 8 weeks (17.1 ± 1.4), whereas that in the placebo group did not improve from 0 weeks (13.7 ± 1.1) to 8 weeks (11.8 ± 1.4); the difference between the 2 groups was significant (*P* < 0.001). In subgroup analysis according to reflux, significant improvements in the LCQ score were observed in the PPI group regardless of reflux (*P* < 0.001 in the reflux group and *P* < 0.001 in the no reflux group, respectively; *P* = 0.188 between the 2 groups). In addition, improvements in LCQ and VAS scores between the standard- and high-dose PPI groups were not significantly different; however, adverse reactions were induced by only the high dose (16.7%).

**Conclusions:**

The results of this pilot study support the empirical use of the standard dose of PPI for 8 weeks in patients suffering from unexplained chronic cough regardless of whether reflux is present.

**Trial registration:**

ClinicalTrial.gov NCT01888549 www.clinicaltrials.gov; cris.nih.go.kr KCT0000543 cris.nih.go.kr/

## Introduction

Chronic cough is a significant health issue affecting 8–12% of adult [1–3], and they experience poor quality of life, and increased healthcare utilization [4,5]. Gastroesophageal reflux disease (GERD) is one of the main etiologies of chronic cough [6], therefore, many guidelines suggest empirical proton pump inhibitor (PPI) therapy [7–9]. However, certain recent studies have revealed disappointing results from PPI therapy [10,11]; therefore, further studies are needed to justify international guidelines widely used in clinical practice. Moreover, the optimal PPI dose has not been clarified. Gastroenterology guidelines recommend the use of the standard dose [12,13]. However, some studies revealed that the a high dose (twice daily) of PPI was superior in treating GERD and extraesophageal symptoms including cough [14]. Therefore, the high dose of PPI is used to treat asthma related to GERD [15,16], and recent studies concerning chronic cough also recommend the high dose of PPI [17,18]. However, this is based on uncontrolled studies and observational data only. The reason why the dose of PPI needed for chronic cough is higher than that in typical GERD is not well explained.

Therefore, we conducted this pilot study to assess the effectiveness of empirical PPI therapy in patients with an unexplained chronic cough, and to determine the most effective PPI dose.

## Methods

### Ethics

This study was approved by the Ethics Committee of the National Evidence-Based Healthcare Collaborating Agency. All protocols were approved by the institutional review board of Gangnam Severance Hospital (3-2011-0103). And this study was registered to www.clinicaltrials.gov (No. NCT01888549) and cris.nih.go.kr (No. KCT0000543). All patients provided written informed consent for participation in the study.

### Study subjects

The subjects were patients between the ages of 19 and 70 years who were admitted to Gangnam Severance Hospital for chronic cough lasting more than 8 weeks, from February 1, 2012 to January 31, 2014 (recruitment period). The last follow up date was April 30, 2014. Exclusion criteria were as follows: subjects suspected to have upper airway cough syndrome (by pulmonary function tests with bronchodilator use and a methacholine bronchial provocation test) [20]; subjects diagnosed with asthma-related cough syndrome or underlying pulmonary disease; current smokers; subjects who had experienced failure of PPI therapy or anti-reflux surgery or procedures; subjects with gastrointestinal tumor or Barrett’s esophagitis; subjects who had experienced upper respiratory infection in the previous 8 weeks; and subjects who were using a PPI, histamine receptor 2 blocker, beta-blocker, angiotensin-converting enzyme inhibitor, corticosteroid, methylxanthine, or anticholinergic at the time of enrollment. All eligible patients who meet the criteria and agree to participate the study were recruited.

### Study flow including PND medication trial

**Fig 1:**
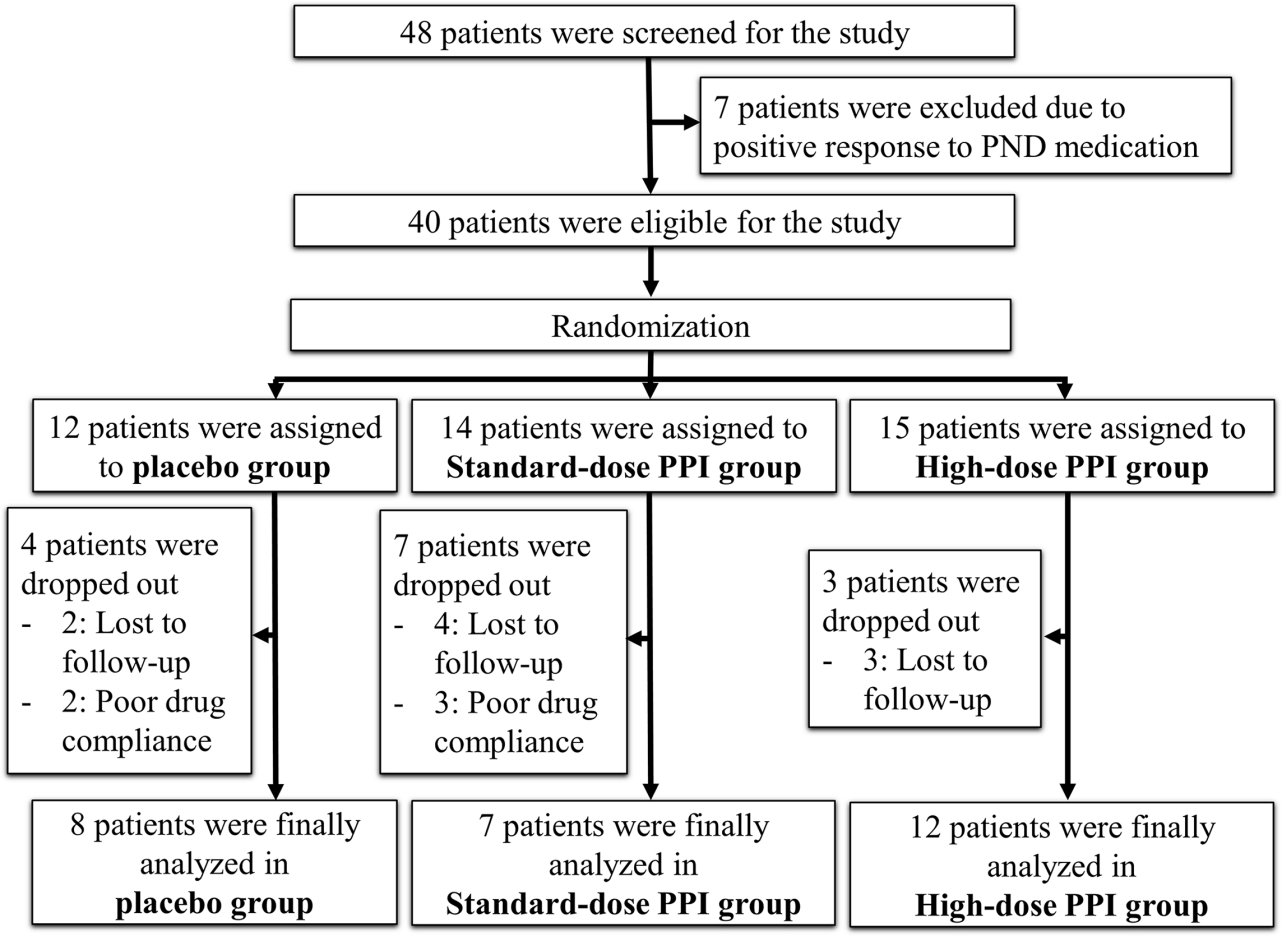
Study flow. ***PND*,** postnasal drip; ***PPI***, proton pump inhibitor.

All 48 participants received postnasal drip (PND) medication, including first generation antihistamines, decongestants, and intranasal steroids, for 2 weeks, and 7 patients were excluded because of a positive response to PND medication. Then, 41 participants were randomized in a blinded fashion using a computerized random number generator (permuted-block randomization) for treatment with placebo, standard-dose PPI, or high-dose PPI at a ratio of 1:1:1. Patients who took <80% of the medication during the study period and participants who were lost to follow-up were excluded from the study. Finally, 8 patients in the placebo group, 7 patients in the standard-dose PPI group, and 12 patients in the high-dose PPI group were enrolled (Fig 1).

### Study intervention and blinding

All subjects were instructed to take their study medication (placebo or esomeprazole 40 mg without identifying features) twice daily, 30 minutes before breakfast and dinner, for 8 weeks. The standard dose of esomeprazole was 40 mg once daily and the high dose was 40 mg twice daily. The randomization method used was permuted-block randomization (block size = 9). Sample sizes were established based on previous studies on chronic cough [18, 19]. Using the expected values based on those studies (4, as an expected difference of mean change; 3, as an expected standard deviation), alpha (0.05), the statistical power (80–90%), the ratio (1:2 as placebo:study), and drop rate (15%), we obtained number of 48, as a minimum total number of subjects for screening in this study.All individuals involved in the direct consultation and treatment of the subjects were blinded to treatment allocation during the study. All work was conducted by the Department of Statistics at our institution.

### Definition of atopy

Atopy was defined as a positive reaction to any major inhalant allergen in skin allergy test (*Dermatophagoides farina*, *Dermatophagoides pteronyssinus*, cockroach, *Alternaria*, cat dander, dog dander, oak, birch, mugwort, and ragweed) [21].

### Cough severity scores

The severity of cough was assessed by visual analogue scale (VAS) and Leicester Cough Questionnaire (LCQ) scores. The LCQ was translated into Korean and has been re-confirmed by Birring and colleagues, who originally developed and validated the LCQ score [22,23]. We defined patients with an LCQ score improvement of more than 2.56 as medication responders [23].

### Reflux tests

The following methods were used to exclude the presence of reflux: (1) esophagogastroduodenoscopy (EGD), (2) 24 h ambulatory esophageal pH/impedance monitoring, and (3) GerdQ questionnaire. If the result of at least 1 of the 3 tests was positive, the patient was determined as to be positive for reflux. With the GerdQ, a diagnosis of GERD was defined by a total score of 8 or more, or 3 or more for impact questions [24].

### Study outcomes

The primary outcomes were changes in the LCQ and VAS scores among groups from baseline to 4 weeks after and 8 weeks after treatment. The secondary outcome was the prevalence of medication responders. Subgroup analysis was planned; and the standard of classification for subgroup was the presence of reflux and the PPI dose.

### Statistics

The Fisher’s exact test was used to assess the significant differences in categorized parameters (distribution of sex, prevalence of underlying diseases, the positivity rate of allergic skin test, the results of 24h pH monitoring, EGD, prevalence of reflux and medication responderbetween the 3 groups. Comparisons of the levels of continuous parameters (all parameters in Table 1 and Table 2 excluding the previously mentioned categorized parameters) between the 3 groups were assessed using the analysis of variance test. Moreover, the significance was rechecked using Bonferroni post hoc testing for significant parameters (age among groups). Significant changes and differences in LCQ and VAS scores according to time and group were analyzed using linear mixed effect models (all the data in figures). In detail, we used a multivariate Gaussian linear model with unstructured covariance. Two fixed effects were included: time and group (both as categorical variables). The possible differences in cough symptom score between groups across time were analyzed according to group*time interactions. These were adjusted for age, sex, hypertension, and atopy; as these 4 values tend to be different among groups (*P*-value > 0.10 and standardized differences > 100.0%). Subgroup analysis was conducted based on the presene of reflux and the PPI dose. All analyses were performed using SPSS software (version 18.0; SPSS Inc., Chicago, IL, USA), and *P*-values of < 0.05 were considered statistically significant.

**Table 1:** Demographic characteristics according to group.

	Placebo (n = 8)	Standard-dose PPI (n = 7)	High-dose PPI (n = 12)	*P*-value	Standardized differences (%)		
					Dp-s	Dp-h	Ds-h
Age (yr)*	**53.1 ± 10.9**	**58.4 ± 6.5**	**38.8 ± 9.6**	**< 0.001†**	**-59.06**	**139.23**	**239.09**
**Sex (female)**	**5 (62.5%)**	**6 (85.7%)**	**4 (26.7%)**	**0.082**	**-54.92**	**77.20**	**147.90**
Height (cm)*	162.0 ± 8.8	158.6 ± 5.1	165.3 ± 7.9	0.189	47.27	-39.46	-100.77
Weight (kg)*	66.0 ± 9.5	63.3 ± 7.0	64.2 ± 8.9	0.819	32.36	19.55	-11.24
Smoking	2 (22.2%)	0 (0.0%)	1 (8.3%)	0.432	75.54	39.41	-42.54
Underlying disease							
**HTN**	**3 (37.5%)**	**0 (0.0%)**	**0 (0.0%)**	**0.031****†**	**109.54**	**109.54**	**0**
DM	1 (12.5%)	0 (0.0%)	0 (0.0%)	0.556	53.45	53.45	0
Laboratory findings*							
WBC (/μL)	6,215.0 ± 1,244.8	7,784.3 ± 2,254.3	8,276.7 ± 2,758.6	0.156	-86.18	-96.34	-19.55
Neutrophil (/μL)	3,587.5 ± 930.3	4,417.1 ± 1,524.9	5,015.8 ± 2,723.5	0.331	-65.68	-70.18	-27.12
Eosinophil (/μL)	178.8 ± 69.2	268.6 ± 244.1	275.8 ± 321.6	0.678	-50.05	-41.70	-2.52
Hemoglobin (g/dL)	14.4 ± 1.3	14.1 ± 1.2	14.9 ± 1.4	0.437	23.98	-37.01	-61.36
Platelet (/μL)	266.3 ± 36.8	310.4 ± 63.9	259.6 ± 46.7	0.100	-84.58	15.94	90.77
**BUN (mg/dL)**	**15.1 ± 3.6**	**14.5 ± 5.1**	**11.3 ± 2.5**	**0.056**	**13.59**	**122.61**	**79.68**
Creatinine (mg/dL)	0.7 ± 0.2	0.8 ± 0.2	0.7 ± 0.2	0.921	-50	0	50
AST (IU/L)	30.3 ± 13.6	26.4 ± 5.5	21.0 ± 6.8	0.098	37.60	86.50	87.32
ALT (IU/L)	27.3 ± 12.2	27.9 ± 15.8	24.5 ± 20.5	0.901	-4.25	16.60	18.58
**Albumin (g/dL)**	**4.6 ± 0.1**	**4.5 ± 0.1**	**4.7 ± 0.2**	**0.092**	**100**	**-63.25**	**-126.49**
Total IgE (KIU/L)	123.4 ± 173.3	63.9 ± 62.2	110.8 ± 87.0	0.678	45.70	9.19	-62.02
Allergic skin test							
**Atopy**	**2 (25.0%)**	**0 (0.0%)**	**6 (54.5%)**	**0.059**	**81.65**	**-63.22**	**-154.78**
PFT*							
FEV_1_ (%)	111.6 ± 17.9	111.3 ± 20.2	101.5 ±11.3	0.290	1.57	67.48	59.88
FVC (%)	106.9 ± 14.3	101.9 ± 18.3	95.0 ± 9.2	0.168	30.45	98.97	47.64
**FEV**_**1**_**/FVC (%)**	**78.8 ± 5.6**	**81.4 ± 2.4**	**83.6 ± 3.3**	**0.042****†**	**-60.35**	**-104.43**	**-76.25**

PPI: proton pump inhibitor; HTN: hypertension, DM: diabetes mellitus; WBC: white blood cells; BUN: blood urea nitrogen; AST: aspartate transaminase; ALT: alanine transaminase; PFT: pulmonary function test; FEV_1_: forced expiratory volume for 1 second; FVC: forced vital capacity; Dp-s: standardized differences between placebo group and standard-dose PPI group; Dp-h: standardized differences between placebo group and high-dose PPI group; Ds-h: standardized differences between standard-dose PPI group and high-dose PPI group

* Data are presented as mean ± standard deviation.

† *P-*value < 0.05 among 3 groups

Bold represents imbalanced variables among groups (*P*-value < 0.10 and standardized differences > 100.0%)

**Table 2:** Reflux-related test results and prevalence of medication responders.

		Placebo	Standard-dose PPI	High-dose PPI	*P*-value
24h pH monitoring					0.357
	N/A	2 (25.0%)	3 (42.9%)	7 (58.3%)	
	Non-acidic	6 (75.0%)	4 (57.1%)	4 (33.3%)	
	Acidic	0 (0.0%)	0 (0.0%)	1 (8.3%)	
EGD					0.797
	N/A	2 (25.0%)	1 (14.3%)	2 (16.7%)	
No GERD		4 (50.0%)	5 (71.4%)	6 (50.0%)	
Minimal GERD		0 (0.0%)	1 (14.3%)	3 (25.0%)	
Definite GERD		2 (25.0%)	0 (0.0%)	1 (8.3%)	
GERD questionnaire		7.9 ± 3.4	5.9 ± 3.9	4.6 ± 3.3	0.159
Reflux		6 (75.5%)	4 (57.1%)	8 (66.7%)	0.876
Medication responder					
	4 weeks	3/8 (37.5%)	3/7 (42.9%)	6/12 (50.0%)	0.888
	8 weeks	2/5 (40.0%)	6/6 (100.0%)	6/7 (85.7%)	0.060

PPI: proton pump inhibitor; N/A: not available; EGD: esophagealgastroduodenoscopy; GERD: gastroesophageal reflux disease

## Results

### Demographics according to group

The mean age of the subjects in the placebo (mean ± standard deviation [SD], 53.1 ± 10.9 years old) and standard-dose PPI (58.4 ± 6.5) groups was significantly greater than that in the high-dose PPI group (38.8 ± 9.6; *P* = 0.008 and *P* = 0.001, respectively, after post-hoc analysis). In the placebo group, the prevalence of hypertension (37.5%) was significantly greater than that in both PPI groups (0.0%; *P* = 0.031). In addition, predominant of male sex and atopy was observed in the high-dose PPI group (*P*-value < 0.10 and standardized differences > 100.0%). Consequently, age, sex, hypertension, and atopy were used for adjustment when comparing cough symptom score trend among groups. Laboratory findings showed that BUN and albumin tended to be lower in high-dose PPI group (*P*-value < 0.10 and standardized differences > 100.0%). Table 1 shows a significant difference in the forced expiratory volume for 1 second (FEV_1_)/functional vital capacity (FVC) ratio (%) among the 3 groups (*P* = 0.042). Other parameters were not significantly different among the 3 groups.

### Reflux-related test results and prevalence of medication responders according to group

Table 2 shows the results of 24 h pH monitoring, EGD, and GERD questionnaire score were not different among the 3groups. Moreover, the prevalence of reflux was also not different among the 3 groups (*P* = 0.876). The prevalence of medication responders at 4 weeks was not different between the 3 groups. The prevalence of medication responders at 8 weeks in the standard-dose PPI group (100.0%) and high-dose PPI group (85.7%) tended to be higher than that in the placebo group (40.0%) (*P* = 0.060). In the pooled analysis, the prevalence of medication responders in the PPI group (92.3%) was significantly higher than that in the placebo group (40.0%; *P* = 0.044) at 8 weeks. In addition, among the medication responders in the PPI group (n = 12), 5 subjects (41.7%) did not show reflux evidence.

### Changes in LCQ and VAS scores according to time and group

**Fig 2:**
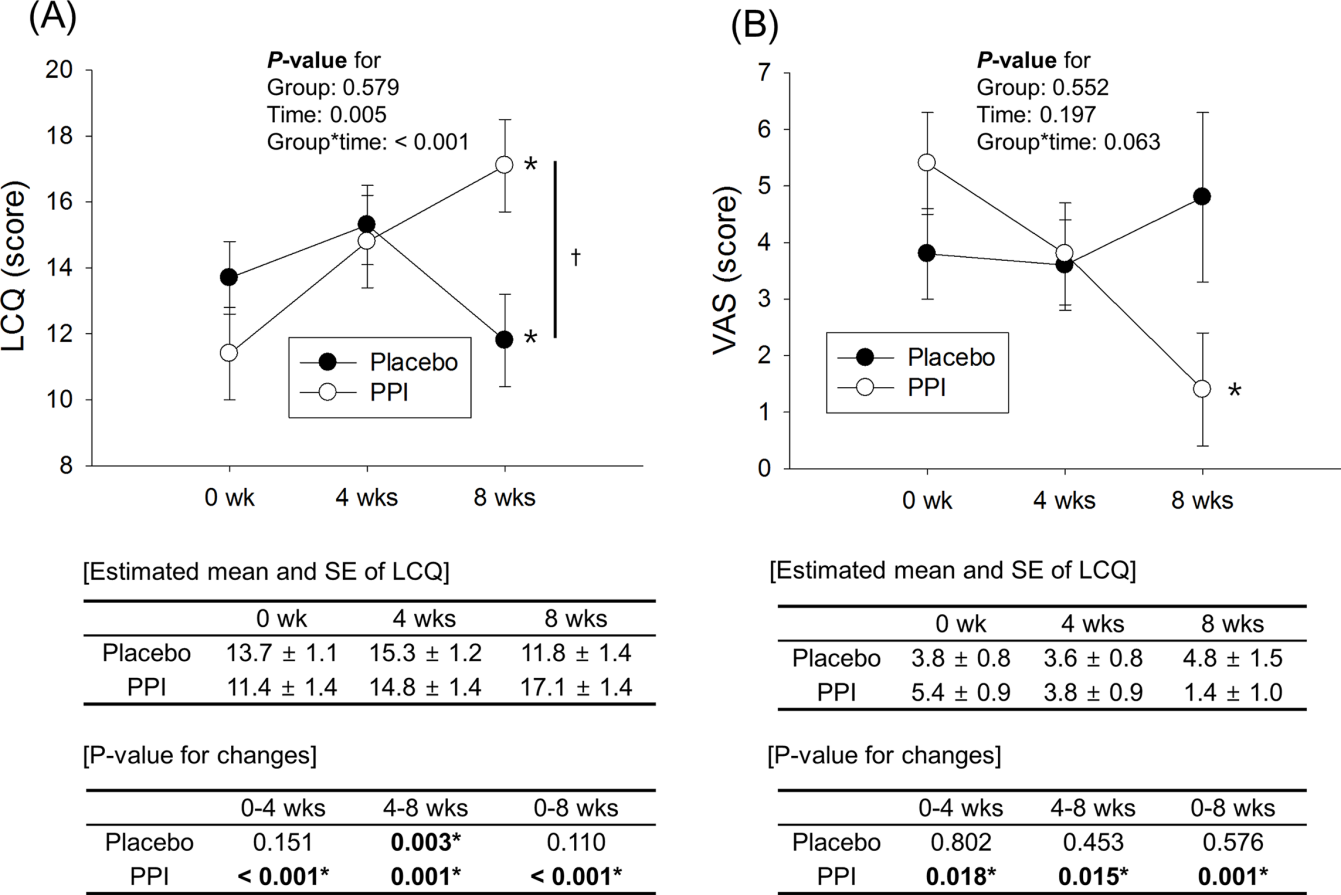
**Change in LCQ (A) and VAS (B) scores according to time and group.** The LCQ and VAS scores are significantly improved through 8 weeks in the PPI group, but not in the placebo group (between-group comparison: LCQ score, *P* < 0.001; VAS score, *P* = 0.063). ***PPI***, proton pump inhibitor; ***LCQ***, Leicester Cough Questionnaire; ***VAS*,** visual analog scale; ***SE***, standard error. * *P*-value < 0.05 according to time; † *P*-value < 0.05 according to time between the 2 groups; obtained by linear mixed model analysis after adjustment for age, sex, hypertension, and atopy.

The LCQ score in the placebo group did not significantly changed from 0 weeks (13.7 ± 1.1) to 4 weeks (15.3 ± 1.2) (*P* = 0.151), but significantly decreased at 8 weeks (11.8 ± 1.4) (*P* = 0.003). Overall, the LCQ score in the placebo group was not improved from 0 weeks to 8 weeks (*P* = 0.110). In contrast, the LCQ score in the PPI group (combined both the standard-dose and high-dose PPI group) significantly improved from 0 weeks (11.4 ± 1.4) to 4 weeks (14.8± 1.4) and to 8 weeks (17.1 ± 1.4; totally, *P* < 0.001). This improvement in the PPI group was significantly different compared with that in the placebo group (*P* < 0.001) (Fig 2A). The symptom change using VAS score showed similar pattern to the results of the LCQ score (Fig 2B).

### Changes in LCQ and VAS scores according to time and reflux

**Fig 3:**
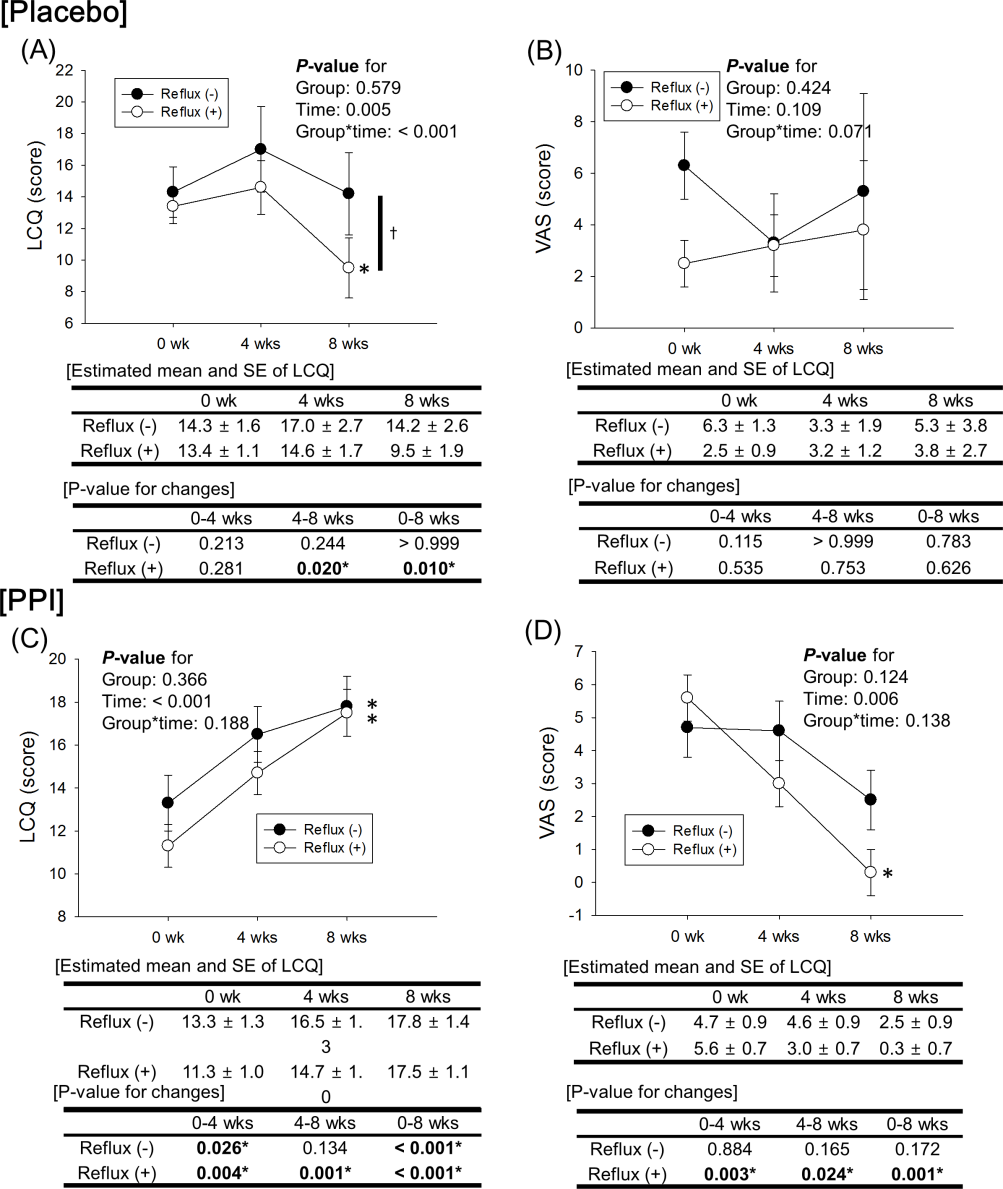
**Change in LCQ and VAS scores according to time and reflux in the placebo group (A, B) and PPI group (C, D).** Significant improvement was seen in the PPI group with and without reflux, but not in the placebo group. ***LCQ*,** Leicester Cough Questionnaire; ***VAS*,** visual analogue scale; ***PPI*,** proton pump inhibitor; ***SE***, standard error. * *P*-value < 0.05 according to time; obtained by linear mixed model analysis after adjustment for age, sex, hypertension, and atopy.

In the placebo group without reflux, the LCQ score did not significantly changed; however, those with reflux showed significantly aggravation of symptoms (*P* = 0.010). The change in LCQ score according to time between the 2 subgroups was significantly different (*P* < 0.001) (Fig 3A). The symptom change using VAS score showed a similar pattern to the results of the LCQ score (*P* = 0.071) (Fig 3B).

In the PPI group, the LCQ score in the reflux subgroup significantly improved from 0 weeks (11.3 ± 1.0) to 4 weeks (14.7 ± 1.0) and to 8 weeks (17.5 ± 1.1) (*P* < 0.001). Even in subjects without reflux, the LCQ score significantly improved from 0 weeks (13.3 ± 1.3) to 4 weeks (16.5 ± 1.3) and to 8 weeks (17.8 ± 1.4) (*P* < 0.001). In addition, there was no significant difference in changes in the LCQ score between the 2 subgroups (*P* = 0.188) (Fig 3C). The symptom change using VAS score were similar to the results of the LCQ score (*P* = 0.138) (Fig 3D).

### Changes in LCQ and VAS scores in the PPI group according to time and dose of PPI

**Fig 4:**
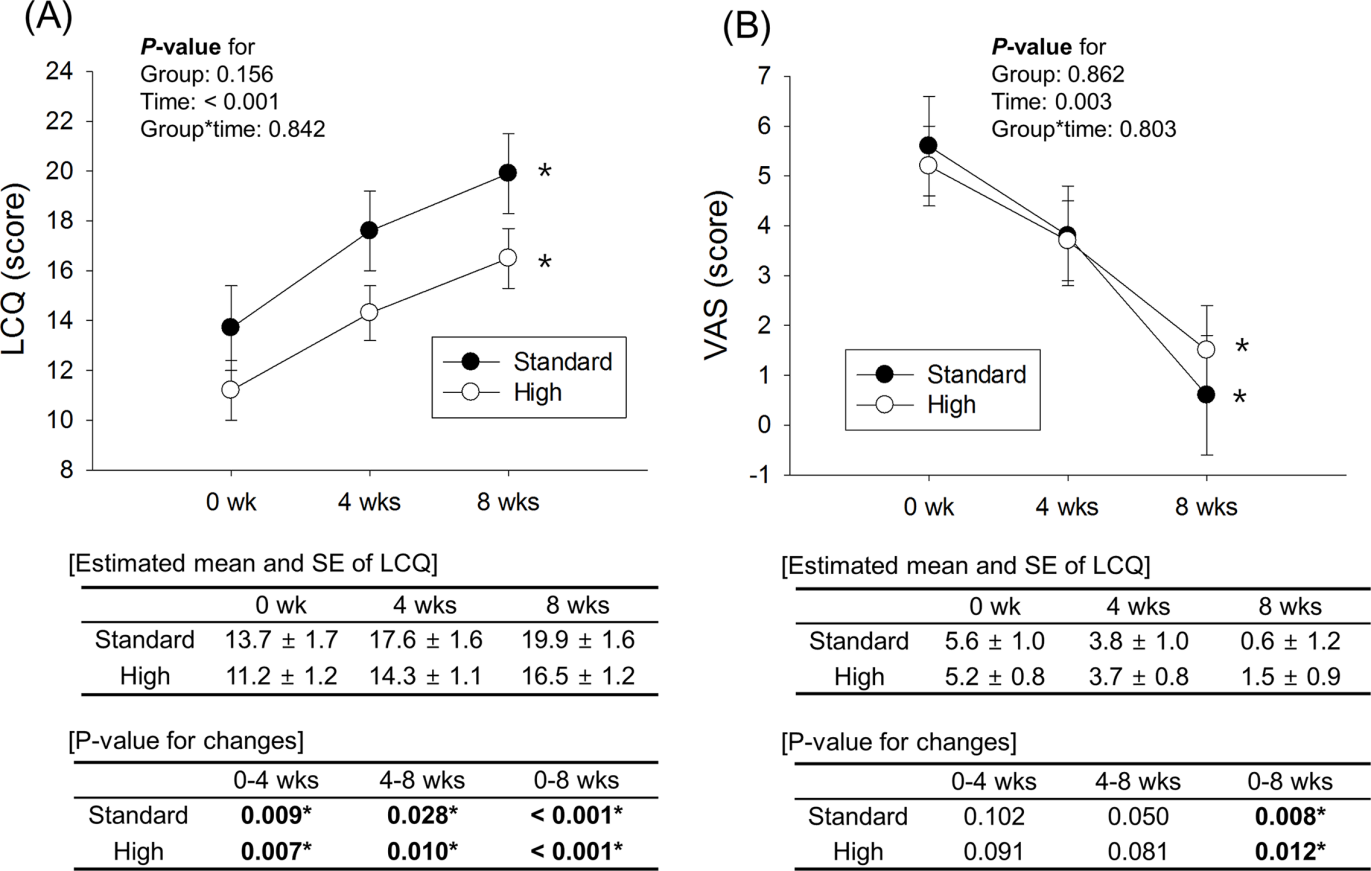
**Change in LCQ (A) and VAS (B) scores according to time and dose of PPI.** Improvement in LCQ and VAS scores is seen in both standard- and high-dose PPI groups, and there is no difference between the standard- and high-dose PPI groups (LCQ score, *P* = 0.842; VAS score, *P* = 0.803). ***LCQ***, Leicester Cough Questionnaire; ***VAS*,** visual analogue scale; ***PPI*,** proton pump inhibitor; ***SE***, standard error. * *P*-value < 0.05 according to time; obtained by linear mixed model analysis after adjustment for age, sex, hypertension, and atopy.

The LCQ score in the standard-dose PPI group (n = 7) significantly improved from 0 weeks (13.7 ± 1.7) to 4 weeks (17.6 ± 1.6) and to 8 weeks (19.9 ± 1.6) (*P* < 0.001), while that in the high-dose PPI group (n = 12) also significantly improved from 0 weeks (11.2 ± 1.2) to 4 weeks (14.3 ± 1.1) and to 8 weeks (16.5 ± 1.2) (*P* < 0.001). In addition, there was no significant difference in LCQ score between the 2 groups (*P* = 0.842) (Fig 4A). The symptom change using the VAS score was similar to that of the LCQ score (Fig 4B).

### Safety and tolerability

Four subjects (14.8%) experienced adverse reactions related to the study drugs. In the placebo group, 1 patient experienced palpitation and another patient experienced urticaria. In the PPI group, 2 patients experienced urticaria, and these patients both were in the high-dose PPI group. In the standard-dose PPI group, no subjects experienced any adverse event.

## Discussion

This double-blind, placebo-controlled, randomized clinical trial revealed significant effects of PPI in patients suffering from unexplained chronic cough. The main finding of this study is that the significant effect of PPI therapy in chronic cough was consistent even in subjects without evidence of reflux. Gastroenterology guidelines recommend acid-suppressive therapy only in patients with chronic cough who have typical symptoms of GERD or objective evidence of GERD [25]. However, historical pulmonology guidelines have recommended an empirical PPI trial in patients with unexplained chronic cough regardless of reflux [7,26]. The significant effects of PPI therapy in patients with chronic cough even without reflux means that those patients might have “silent” reflux [27]. Among the medication responders in the PPI group, 41.7% did not show reflux evidence. This 41.7% of patients might be defined as those showing “silent” reflux. In this study, the diagnosis of “reflux” was highly sensitive; that is, negative reflux in this study meant absolute negative status regarding observed and unobserved reflux, because of the wide definition of the term “reflux.” We reconfirmed that chronic cough can be the only presenting symptom of GERD, in agreement with previous literature [28,29]. However, some researchers and guidelines in the western countries still suggest that PPIs should be prescribed only in chronic cough accompanied by GERD symptoms [30]. This might be because the prevalences of obesity and “silent” reflux differ between Asia and the West [31]. The lower prevalence of obesity may induce a higher prevalence of “silent” reflux, which might lead to superior effects of PPIs in Asia than the west.

Cough receptors are also present in the distal esophagus [32], therefore, any significant stimuli in the distal esophagus can induce a cough through various mechanisms [33,34]. GERD is the main cause of chronic cough, so many guidelines recommend PPI therapy in the management of chronic cough. However, scientific evidence remains insufficient [9,35,36]. Some studies have shown significant effects of PPI therapy [19,37]. However, other studies have yielded disappointing results [18,35]. Two recent placebo-controlled, randomized trials concluded that PPIs are not beneficial. Faruqi et al. showed that there was no significant difference in LCQ score improvement between placebo and PPI groups. However, when we looked more closely, the PPI group had a significant improvement in the LCQ score, whereas the placebo group showed negative results. Shaheen et al. also found a significant improvement of cough symptom in the PPI group; however, significant differences between the placebo and PPI groups were not shown. In summary, although those 2 previous studies concluded that results are negative, they suggest potential benefits of PPI therapy in unexplained chronic cough. In addition, the difference in design between those studies from ours might lead to different results. Shaheen et al. included subjects presenting with a chronic cough and with no obvious etiology and excluded subjects with reported heartburn symptoms. Faruqi et al. designed the intervention group to receive esomeprazole 20 mg twice daily, whereas guidelines recommended esomeprazole 40 mg twice daily.

Our study has several strengths. First, all subjects underwent a therapeutic trial for PND prior to randomization to exclude PND syndrome, which cannot easily be detected by history, physical examination, paranasal sinus radiography, or laryngoscopy. Our PND medication trial before randomization might have led to a more accurate assessment of the effects of PPIs on unexplained chronic cough. Second, we included standard and high-dose of PPI groups. Until now, the dose of PPI to treat chronic cough has remained controversial. Some studies recommended the a high dose [19,37]; however, these previous studies did not include a standard-dose arm. The present study showed for the first time that the standard dose of PPI is sufficient and safe to manage chronic cough. Third, this study showed an ongoing improvement in the symptoms of chronic cough at 4 and 8 weeks of therapy which is similar to previous literature [9]. Therefore, we suggest that at least 8 weeks is needed to obtain sufficient effects. Last, we made a concerted effort to reflect real-world clinical practice situations; all available diagnostic evaluation techniques for possible contributing factors for chronic cough were performed, and then empirical PPI therapy was administered with or without reflux. The high- and standard-dose PPI design also took into account the real-world situation.

This study has some limitations. We included a small number of patients. Owing to the nature of chronic cough, enrollment and maintenance of this type of study is difficult. Subjects usually consider chronic cough a simple symptom, not a disease. Subjects frequently show poor compliance and finally drop out regardless of whether they feel an improvement in chronic cough. Therefore, we could enroll only a small number of subjects; moreover, approximately 30% of the enrolled patients were excluded. However, the results including these patients would have been more unreliable because of their extremely poor compliance (minimum compliance value = 58.1%). For these reasons, we could not conduct an intention-to-treat analysis. Thus, this study should be considered a pilot study. Second, the treatment duration was only 2 months. The relatively short duration of this study was the longest duration possible to continue the study under a real-world situation. Some studies conducted for 2 months obtained similar results to those of this study [19,38]; thus, we considered the study duration of 2 months to be sufficient.

## Conclusions

This pilot study showed significant benefit from PPI therapy in patients with unexplained chronic cough regardless of whether reflux evidence was evident. The standard dose of PPI for more than 8 weeks is safe and effective. This supports many guidelines formed on the basis of expert opinion that have not been substantiated by sufficient scientific evidence.

## Supporting information

S1 Document Protocol of this study. (DOC)

S2 Document Checklist for CONSORT 2010. (DOC)
