# Supplementary material for: Effectiveness of proton pump inhibitor in unexplained chronic cough
Source: PLoS One. 2017 Oct 10;12(10):e0185397. doi: 10.1371/journal.pone.0185397 (PMC5634560; doi:10.1371/journal.pone.0185397)
Supplement: S1 Document — (DOC) [file pone.0185397.s001.doc]

**Research Proposal**

**1. Research Title**

A double-blind, placebo-controlled, randomized controlled trial on the efficacy of proton pump inhibitors in patients with chronic cough due to gastroesophageal reflux disease.

**2. Overview of Research Requirements**

Cough is one of the most common symptoms that prompt patients to visit a hospital, and is experienced by 14 to 23% of non-smokers. Cough is classified into different subtypes based on its time period. Acute cough persists below 3 weeks, subacute cough persists within 3 to 8 weeks, and chronic cough persists over 8 weeks. In the case of chronic cough, symptoms only improve if the exact cause of the disease is identified and treated properly. It has been reported that postnasal drip syndrome, asthma, and gastroesophageal reflux disease (GERD) were the most important causative diseases ^1-3^.

In addition to chronic cough, other respiratory symptoms associated with GERD include hoarseness, sore throat, nighttime inhalation, asthma symptoms, chest pain, and sleep apnea. Upper GERD accounts for 20 to 40% of patients with chronic cough. It is reported that in 50–75% of patients, chronic cough is the only GERD-associated symptom without other symptoms such as reflux, heartburn, sourness, and chest pain ^4-6^. It has been reported that 50% of patients with chronic cough without a clearly identified cause such as bronchial asthma or post-nasal drip syndrome, have gastroesophageal reflux.

Proton pump inhibitor (PPI) is widely recommended for the treatment of chronic cough due to GERD^7^, but there is little evidence to support it. To date, most prospective studies on the role of PPI in the treatment of chronic cough due to GERD have yielded ambiguous results. The reasons for that include the small number of the study population and the lack of using a verified objective measurement tool to evaluate the therapeutic effect of PPI. Moreover, the degree of symptom improvement was weak in most previous reports ^8,9^. Although the 24-hour pH monitoring test has been performed in many studies, it is limited in predicting the response to treatment of GERD and is not effective in detecting other potential causes of GERD such as non-acid or weakly acid reflux disease. Thus, it is recommended to perform impedance acidity test with the 24-hour pH monitoring test to ensure accurate detection and differentiation of GERD.

Despite the lack of reliable clinical data, the Clinical Practice Guideline has been recommending empirical PPI administration in patients with chronic cough due to GERD^7^. However, recent Cochrane analysis^10^ and meta-analysis ^11^ suggested the need for a randomized controlled trial (RCT) using appropriate cough assessment indicators to justify empiric PPI treatment in international clinical guidelines. Regarding the appropriate treatment dosage of PPI, a recent study reported that treatment of chronic cough due to GERD is most effective with dosage that is twice the standard dose ^12^. Even the guideline used in Korea for clinical management of GERD ^13^ recommends doubling the dosage of PPI if the standard dose is not effective; however, this statement lacks evidence. The therapeutic effect of PPI was better in acidic reflux disease compared to non-acidic (weakly acidic) reflux disease, but mild improvement of certain symptoms was observed in in non-acidic (weakly acidic) reflux disease ^14^. This needs to be verified with appropriate study.

In a previous study, Shaheen et al. ^18^ prospectively analyzed the results of treatment with high-dose PPI for 12 weeks in patients with chronic cough due to non-acidic (weakly acidic) reflux disease without typical GERD symptoms such as heartburn. Patients with chronic cough due to non-acidic (weakly acidic) reflux disease did not have significant improvement in the frequency or severity of cough symptoms or quality of life during the 12-week period of high-dose PPI treatment. This result argues against the guideline recommendation of prophylactic treatment with PPI in patients with chronic cough. However, this study has several limitations. The sample size of the control and treatment group was too small to have statistical influence. It is difficult to conclude whether treatment was ineffective only in patients with non-acidic (weakly acidic) reflux disease, because this study was conducted only in patients who are not generally treated with PPI, without comparative analysis on patients with chronic cough due to GERD or therapeutic effect with standard dose of PPI.

Birring, the author of the Leicester Cough Questionnaire (LCQ), reported in a recent issue of the Journal of the International Society for Thoracic & Cardiovascular Surgery entitled, "Evaluation and Treatment of Chronic Cough" that there are no randomized controlled trials that reproduce high success rates that have been shown in previous small studies with PPI treatment in chronic cough ^19^. He emphasized the need for further studies. Therefore, we aimed to compare the efficacy of high-dose PPI, standard dose PPI, and placebo-controlled treatment in patients with chronic cough due to GERD and non-acidic (weakly acidic) reflux disease. We obtained the original author’s permission to use the LCQ, which is an objective evaluation index for coughing, in this study, and we will use the translated Korean version of the questionnaire for the first time. We expect to identify a more concrete correlation between cough symptoms and GERD by performing both impedance acidity test and the conventional 24-hour pH monitoring test.

**3. Research Purpose**

We aim to verify the efficacy of PPI treatment in patients with chronic cough due to GERD using a double-blind, placebo-controlled, randomized controlled trial. The purpose of this study is to compare therapeutic effects of PPI in patients with GERD to those with non-acidic (weakly acidic) reflux disease. We will divide the treatment group into high-dose PPI group and standard-dose PPI group to compare with the placebo control group. Evaluation of therapeutic effect will be performed two times, at 4 weeks and 8 weeks of treatment.

In conclusion, we will investigate whether high-dose PPI treatment in patients with chronic cough due to GERD is superior compared to those who receive standard dose of PPI treatment. In addition, we aim to investigate whether treatment with PPI results in improvement of symptoms or quality of life in patients with chronic cough due to non-acidic (weakly acidic) reflux disease. We plan to present evidence that recommends an appropriate treatment time period.

**4. Research Place and Periods**

The research will be conducted at the *Gangnam Severance Hospital of Yonsei University College of Medicine* over a period of *24 months* from the date of initial IRB approval.

**5.. Participants selection criteria and screening test items**

The selection criteria for the study participants were adult male and female patients between the ages of 18 and 70 years who visited the hospital with complaint of chronic cough for more than 8 weeks. Patients who met the following criteria were excluded: (1) aged under 18 or over 70 years; (2) current smokers or have smoked within the last 3 months; (3) underlying lung disease (including those with evidence of radiologic exposure); (4) bronchial asthma (those who are currently being treated or those who have been diagnosed with pulmonary function test and bronchodilator tests after admission); (5) post-nasal syndrome (patients who are currently undergoing treatment, or diagnosed with physical examination and radiographic findings, or clinically diagnosed with improvement in cough symptoms after administration of antihistamine-nasal decongestant medication); (6) previously failed treatment with PPI; (7) have previously undergone surgical or endoscopic anti-reflux treatment; (8) history of gastrointestinal tumors or Barrett’s esophagitis; (9) upper respiratory infection within the last 8 weeks; and (9) patients currently taking PPI, H2 inhibitors, beta blockers, angiotensin converting enzyme inhibitors, corticosteroids, methyltestosterone, or anticholinergics.

**6. Target number of participants and basis of calculation**

The target number of participants to be included in the study is 60. Based on the consultation with Gangnam Severance Statistics Department, the effective number of participants per group was 15 patients. Each group needed 20 participants. Considering the potential drop-out rate of 10%, a total of 60 people (20 per group) will be recruited.

*Statistical advisory: The statistical advisors are (1) the assistant professor Lim Hyun-sun, Kangnam Clinical Medical Research Center and (2) Dr. Surinder S Birring, Department of Respiratory Medicine, King's College Hospital, London, UK (02032994630, email:* [*surinder.birring@nhs.net*](mailto:surinder.birring@nhs.net)*).*

**7.. Research design and methods**

Patients who visited the outpatient department of respiratory medicine at Gangnam Severance Hospital for chronic cough will be included in the study. Patients with typical GERD symptoms such as acid reflux, heartburn, sourness, and chest pain, and patients with presumed non-acidic (weakly acidic) reflux disease without symptoms of asthma or post-nasal drip syndrome are targeted ^15^.

Using a randomized, double-blinded method, patients eligible for the study will be assigned to high-dose PPI treatment group, standard-dose PPI treatment group, and placebo control group. All participants will be evaluated with LCQ and Cough VAS 4 and 8 weeks after treatment (Figure 1).

The results of PPI treatment in patients with GERD will be compared according to the duration of treatment and dosage of PPI used. In subgroup analysis, the therapeutic effect of PPI in patients with typical GERD will be compared to its effect in patients with atypical non-acidic (weakly acidic) reflux disease.

7-1. Participants selection criteria

Among patients with chronic cough, the patients suspected to have gastroesophageal reflux will be selected based on history, radiologic finding, and endoscopic examination. Based on sinus radiography and physical examination, patients with post-nasal drip syndrome will be excluded from the study. Based on pulmonary function test, bronchodilator test, and blood test, patients with bronchial asthma will also be excluded. In patients without identifiable causes of chronic cough, antihistamines and nasal decongestants will be administered for 1–2 weeks with follow-up assessment to evaluate the risk of post-nasal drip syndrome, which commonly exists without identifiable abnormalities on exams.

If gastroesophageal reflux disease is suspected, the following tests will be performed in the department of gastroenterology. Upper gastrointestinal endoscopy is performed to confirm the presence of esophagitis and mucosal lesions other than esophagitis. A 24-hour ambulatory esophageal pH monitoring is performed to measure the acidity of distal esophagus and to confirm the presence of pathological gastric acid reflux and its association with reflux symptoms. The esophageal impedance test is performed to confirm the presence of non-acidic (weakly acidic) reflux disease and its relation to reflux symptoms.

However, patients with chronic cough without diagnosis of asthma or post-nasal drip who did not undergo gastrointestinal endoscopy, 24-hour pH monitoring, or impedance test are still not completely excluded from the study because they are more likely to have chronic cough due to GERD. Based on the presence or absence of GERD symptoms, these patients will be divided into GERD group and non-acidic (weakly acidic) reflux disease group.

7-2. Patients registration

The research will be conducted on patients with chronic cough without any symptomatic improvement after treatment with antihistamine and nasal decongestants. After signing the agreement form, each patient will be assigned a unique 3-digit participant number. Each number will be assigned in an ascending order starting with the smallest number, and all participants will be identified using this number throughout the duration of the trial.

Patients will be randomly assigned to a high-dose PPI treatment group, a standard-dose PPI treatment group, and placebo control group.

For the double-blinded process, a randomized table provided by the Clinical Trial Center will be submitted to the Clinical Pharmacy. The physician will prescribe a single code to the patient and the clinical pharmacy will provide the drug corresponding to the single code on the random number table.

*Random number assignment designer: Chung Hyun Choi, Clinical Trials Center, Yonsei University.*

*The test drug (Esomeprazole, Esomezol®) and placebo made in the same form as the test drug will be provided by the grant funding agency.*

**Figure 1. Flow chart of the participants**


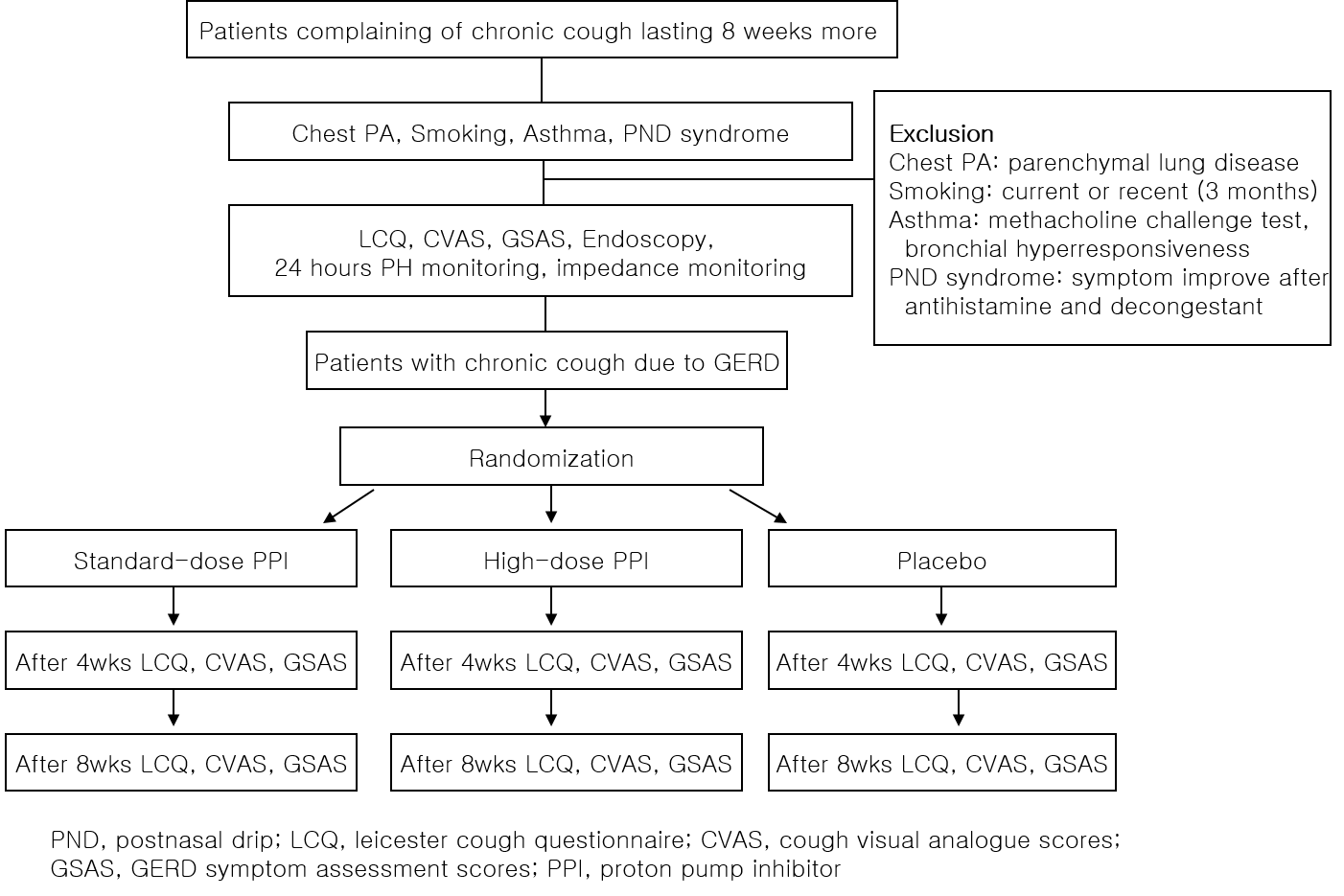


7-3. Clinical trial evaluation

The total period of the treatment will be 8 weeks.

1. Pre-test evaluation:

The pre-test evaluation is replaced by the results observed in previous examinations.

| Evaluation items | | Time before the first administration |
| --- | --- | --- |
| 1. Participants agreement | Obtain | Upon registration |
| 2. History and physical examination | History - Adjuvant medication use, allergies, peptic ulcer disease and reflux esophagitis, basal lung disease, congenital disease, smoking history  Physical examination- height, weight, existing symptoms | Prior to screening |
| 3. Hematologic test | Hemoglobin, hematocrit, leukocytes, neutrophils/eosinophils, platelets | Prior to screening |
| 4. Immunological test | IgE | Prior to screening |
| 5. Biochemical examination | Chemistry profile | Prior to screening |
| 6. Pregnancy test^**^ (those applicable) | Pregnancy test stick | During registration or during pregnancy visit |
| 7. Radiologic evaluation | Chest X-ray and P.N.S  Other tests if clinically necessary | Prior to screening |
| 8. Pulmonary function test | PFT, BD or M-test | Prior to screening |
| 9. Gastrointestinal system examination | Upper gastrointestinal endoscopy, 24 hour pH test, impedance pH test | Prior to screening |

^**^Urine or serum test may be performed depending on need. At the time of visit, pregnancy stick strip will be used for pregnancy test in female subjects whose menstrual period is over or those within period of possible pregnancy after LNMP check.

1. Evaluation during treatment

| Evaluation items | | Time period |
| --- | --- | --- |
| 1. Physical assessment | Symptoms and signs | At the time of random assignment, after 4 weeks, and after 8 weeks |
| 2. Evaluation of treatment effect | Cough VAS, LCQ | At the time of random assignment, after 4 weeks, and after 8 weeks |
|  | GSAS | At the time of random assignment, and after 8 weeks |
| 3.Adverse effects** |  | See below |
| **The observation period for adverse reaction will be from the first date of drug administration to the final date. Serious adverse reactions should be followed as specified in the protocol. | | |

**8. Observation items: Clinical examination items and observation methods**

The observation items will include age, sex, smoking history, underlying disease, cough analogue index, Lester cough questionnaire, and gastroesophageal reflux disease symptom evaluation index

The clinical exam items will include chest X-ray, sinus radiography, CBC, IgE, GOT/GPT, total bilirubin, albumin, CRP, BUN/Cr, GFR, pulmonary function test (bronchodilator test, methacholine challenge test), upper gastrointestinal tract endoscopy, 24-hour pH monitoring, Impedance test

|  | ^*^Visit 0 | ^*^Visit 1 | ^*^Visit 2 | Visit 3 | Visit 4 |
| --- | --- | --- | --- | --- | --- |
|  | screening | enrollment | randomization | After 4 weeks | After 8 weeks |
| Selection/exclusion criteria | X | X |  |  |  |
| Disease history/physical examination | X |  |  |  |  |
| Patient agreement | X |  |  |  |  |
| Pregnancy test | X |  |  |  |  |
| Before trial/medication | X |  |  |  |  |
| Symptoms and signs | X | X | X | X | X |
| LCQ |  |  | X | X | X |
| Cough VAS |  |  | X | X | X |
| GSAS |  |  | X |  | X |
| Adverse reaction |  |  | X | X | X |
| Clinical drug administration/return |  |  | X | X | X |
| Compliance |  |  |  |  | X |

^*^Assessments for visit 0, 1, 2 can be completed in one day.

**9. Predicted Side Effects and Usage Precautions**

9-1. Adverse reactions

1. The following adverse reactions were confirmed or suspected during the clinical trials and post-marketing investigations. There was no correlation with dosage. Adverse reactions were classified based on the frequency (Frequently > 1/100, < 1/10; Not Frequently > 1/1,000, < 1/100; Rarely > 1/10,000, < 1/1,000; Very rarely < 1/10,000).

- Blood and lymphatic system: rarely leukopenia, hypoplasia, very rarely agranulocytosis, pancytopenia

- Immune system: rare reactions such as fever, angioedema, anaphylactic reaction/shock

- Metabolism/nutrition: rare peripheral edema, rare hyponatremia

- Psychiatry: infrequent insomnia, rare agitation, mental confusion, depression, very rare aggressive behavior, hallucinations

- Nervous system: frequent headache, infrequently dizziness, drowsiness, rare tasting disorder

- Visual system: rare blurriness

- Auditory system: infrequently dizziness

- Respiratory system: rare bronchospasm

- Gastrointestinal system: pancreatitis, frequent abdominal pain, constipation, diarrhea, nausea/vomiting, infrequently mouth dryness, stomatitis, candida esophagitis

- Hepatobiliary system: infrequently elevated liver enzymes, rare hepatitis with or without jaundice, very rare hepatic failure, or encephalopathy in patients with existing liver disease

- Skin and Subcutaneous Tissue: infrequently dermatitis, pruritus, rash, urticaria, rare hair loss, light sensitivity, very rare polymorphic erythema, Stevens-Jones Syndrome, toxic epidermal necrosis (some were fatal)

- Musculoskeletal system: rare arthralgia, muscular pain, very rare muscle weakness

- Kidney and urologic system: very rare interstitial nephritis

- Reproductive system: very rare gynecomastia

- Whole body and administration site: rare asthenia, hyperhidrosis

1. The following adverse events were reported to be associated with or likely to be associated with this drug at an incidence of less than 1%.

- Body: Abdominal distension, allergic reaction, back pain, chest pain, facial edema, facial flushing, fatigue, fever, flu-like disorder, general edema, leg edema, boredom, pain, stiffness

- Cardiovascular system: redness, hypertension, tachycardia

-Endocrine system: thyroid disorder

- Gastrointestinal system: irritable bowel syndrome, constipation, indigestion, dysphagia, gastrointestinal disorder, upper abdominal pain, bloating, esophageal disorder, frequent bowel movements, gastroenteritis, gastrointestinal bleeding, hiccups, black stools, oral disorders, pharyngeal disorders, rectal disorders, increased gastrin level, tongue disorders, tongue edema, ulcerative stomatitis, vomiting.

- Auditory system: earache, tinnitus

-Hematologic system: Anemia, hypochromia, cervical lymphadenopathy, epistaxis, leukocytosis

-Liver: bilirubinemia, liver dysfunction

-Metabolism/Nutrition: diabetes, hyperuricemia, hyponatremia, increased alkaline phosphatase, obesity, vitamin B12 deficiency, weight gain, weight loss

- Musculoskeletal: arthritis, arthralgia, muscle cramps, fibromyalgia, hernia, rheumatoid arthritis

- Nervous system / psychology: Anorexia, anxiety, increased appetite, confusion, aggravation, depression, hyperactivity, irritability, depression, erectile dysfunction, insomnia, migraine, tension headache, sleep disorder, tremor, dizziness, visual field defect

- Reproductive system: Dysmenorrhea, menstrual disorders, vaginitis

- Respiratory system: asthma exacerbation, cough, dyspnea, pharyngitis, rhinitis, sinusitis

- Skin / Appendages: Acne, anal pruritus, redness, erythema, spots

- Special senses: otitis, anosmia, loss of taste

-Urinary tract: urinary abnormalities, albuminuria, cystitis, dysuria, fungal infections, hematuria, candidiasis, genital candidiasis

- Visual: conjunctivitis, visual anomalies

- Endoscopic abnormalities: duodenitis, esophagitis, esophageal stricture, esophageal ulcer, esophageal varices, gastric ulcer, gastritis, bleeding, benign tumors, Barrett's esophagus, mucosal discoloration

9-2. General Cautions

1. Because this drug may mitigate the symptoms or delay the diagnosis of malignant tumors, a test should be performed to confirm the presence of malignancy if there is a suspicion of malignant gastric tumor with alerting symptoms (unintended significant weight loss, recurrent vomiting, dysphagia, hemoptysis, or chest pain).
2. Patients taking this drug for a long period (especially more than one year) should be tested regularly.
3. Patients requiring this drug as needed should be advised to consult their physician when there is a change in their symptoms. If the drug is prescribed to be given as needed, interaction with other drugs due to changes in blood concentration of this drug should be considered.
4. When prescribing or administering this drug for Helicobacter pylori eradication therapy, interaction with other medications should be considered. Clarithromycin is a potent CYP3A4 inhibitor, so contraindications and drug-interaction of clarithromycin should be considered in co-administration with drugs that are metabolized by CYP3A4 such as cisapride.
5. Administration of proton pump inhibitors may slightly increase the risk of gastrointestinal tract infection by Salmonella and Campylobacter.
6. In some international epidemiological studies, proton pump inhibitor therapy has been reported to be associated with increased risk of hip, wrist, and vertebral fractures. The risk of fracture was increased in patients receiving higher doses above recommended doses and those with long-term PPI use over 1 year.
7. This medicine contains white sugar. Patients with rare genetic disorders such as fructose intolerance, glucose-galactose malabsorption, or sucrose-isomaltase deficiency should not take this drug (only with saccharide-containing preparations).

9-3. Administration during pregnancy and breastfeeding

1) Administration during pregnancy

There are no clinical data on exposure to this drug during pregnancy. Animal experiments did not reveal any direct or indirect adverse effects related to development of embryo / fetus. Animal studies of racemic mixtures did not show direct or indirect adverse effects of this drug on pregnancy, labor, or postpartum development. However, this medicine should be prescribed with caution to pregnant women.

2) Administration during breastfeeding

It is not known whether this drug will be released into human milk. No experiments were conducted on breastfeeding subjects. Therefore, this drug should not be administered during breastfeeding due to limited evidence.

9-4. Interaction

1. Due to the decrease in gastric acidity during the administration of this drug, the absorption of drugs that depend on acidic environment may decrease or increase. Similar to other inhibitors of acid secretion or antacids, the administration of this drug may reduce the absorption of ketoconazole and itraconazole.
2. This drug is metabolized by CYP2C19 and CYP3A4. Also, this drug inhibits CYP2C19, which is the main metabolic enzyme of this drug. When co-administered with a drug that is metabolized by CYP2C19, such as diazepam, citalopram, imipramine, clomipramine, phenytoin, and warfarin, it may be necessary to reduce the dosage of these since their plasma concentrations may increase. The combined use of CYP2C19 substrate diazepam and 30 mg of this drug reduced diazepam clearance by 45%. In patients with epilepsy, the plasma level of phenytoin increased by 13% when phenytoin was co-administered with 40 mg of this drug. Plasma concentrations of phenytoin should be monitored at the beginning or upon discontinuation of this drug. Omeprazole (400 mg once daily) increased the Cmax and AUCτ of the boliconazole (CYP2C19 substrate) by 15% and 41%, respectively.

In patients receiving warfarin in clinical trials, administration of 40 mg of this drug resulted in coagulation time within the normal range. However, post-marketing investigations have reported several clinically significant elevations in INR during concomitant administration of both drugs. Therefore, monitoring is required when starting or stopping concomitant use of warfarin or other coumadin derivatives with this drug.

1. Concomitant use of omeprazole (20 mg once daily) and Atazanavir 300 mg / ritonavir 100 mg in healthy volunteers resulted in a decrease in Atazanavir exposure (AUC, Cmax, Cmin decreased by about 75%). Atazanavir 400 mg four times a day cannot counteract the effects of omeprazole on atenazabir exposure. Proton pump inhibitors including this drug cannot be co-administered with Atazanavir.
2. Administration of clarithromycin (500 mg bid), a CYP3A4 inhibitor, increases the AUC of this drug by a factor of two. Concomitant use of CYP2C19 and CYP3A4 inhibitors with this drug may increase the exposure of this drug by more than a factor of two. A CYP2C19, CYP3A4 inhibitor, Boniconazole, increased the AUCτ of omeprazole by 280%. Because of this, there is no need to adjust the dose of this drug. However, dose adjustment should be considered in patients with severe hepatic dysfunction and in patients requiring long-term PPI therapy.
3. In healthy volunteers, combined administration of cisapride and 40 mg of this drug resulted in a 32% increase in AUC and 31% increase in half-life, but there was no significant increase in peak plasma concentration. These interactions did not alter the effect of cisapride on cardiac electrophysiology. In other words, the QTc interval was not further prolonged by the combined administration of cisapride and this drug, other than the slight prolongation of the QTc interval reported with cisapride use alone.
4. This drug did not have clinically significant effect on the pharmacokinetics of amoxicillin or quinidine.
5. A short-term study on the combination of naproxen and rofecoxib did not reveal clinically significant pharmacokinetic interactions.

**10. Criteria for Suspending/Withdrawing the Clinical Research**

1. Patient withdraws consent or requests treatment discontinuation
2. Insufficient treatment effect
3. Use of another inhibitors during the clinical trial period
4. The physician judges that the advancing clinical research is impossible due to the disease progression
5. Violation of the research plan or test requirements
6. Lack of follow-up and timely evaluation

The cause (e.g., death and lack of follow-up) and date of withdrawal for all patients who dropped out of the study should be noted in the case record. The physician will be required to complete all testing procedures at the time the test is discontinued.

**11. Evaluation Criteria, Method, and Analytical Method (Statistical Analysis Method)**

11-1. Validated Evaluation Criteria

In order to verify the therapeutic effect on chronic cough, a cough assessment tool that can objectively evaluate the severity of cough is essential.

1. The most commonly used assessment tool in clinical studies is the cough visual analogue score (cough VAS) ^16,17^. This is a subjective assessment of the patient's own experience by correlating the symptoms with a score on a 10-cm ruler that is marked "no cough" at one end and "most severe cough" at the other end. This assessment has a reactive and recurrent advantage, but it has the disadvantage of being impossible to compare the severity of cough between individuals or between groups.


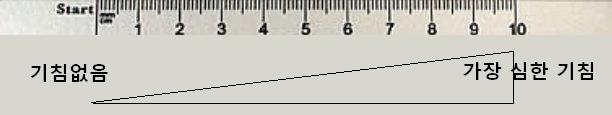


1. Another measure is the Leicester cough questionnaire (LCQ), which is a questionnaire on the quality of life related to cough ^16^. It consists of 19 items in three categories made up of physical, psychological, and social characteristics. The evaluation table scores each item out of 7 points. LCQ has been used in many cough-related studies that have validated its objectivity. As a result, we have already received permission to use the original LCQ for this study. We will consult a bilingual translator to translate the original LCQ into Korean, and then consult another translator to translate it back to English. We will then send the re-translated English version of LCQ to the original authors for review and approval. A series of standardized translations will be used to complete the translation of LCQ.


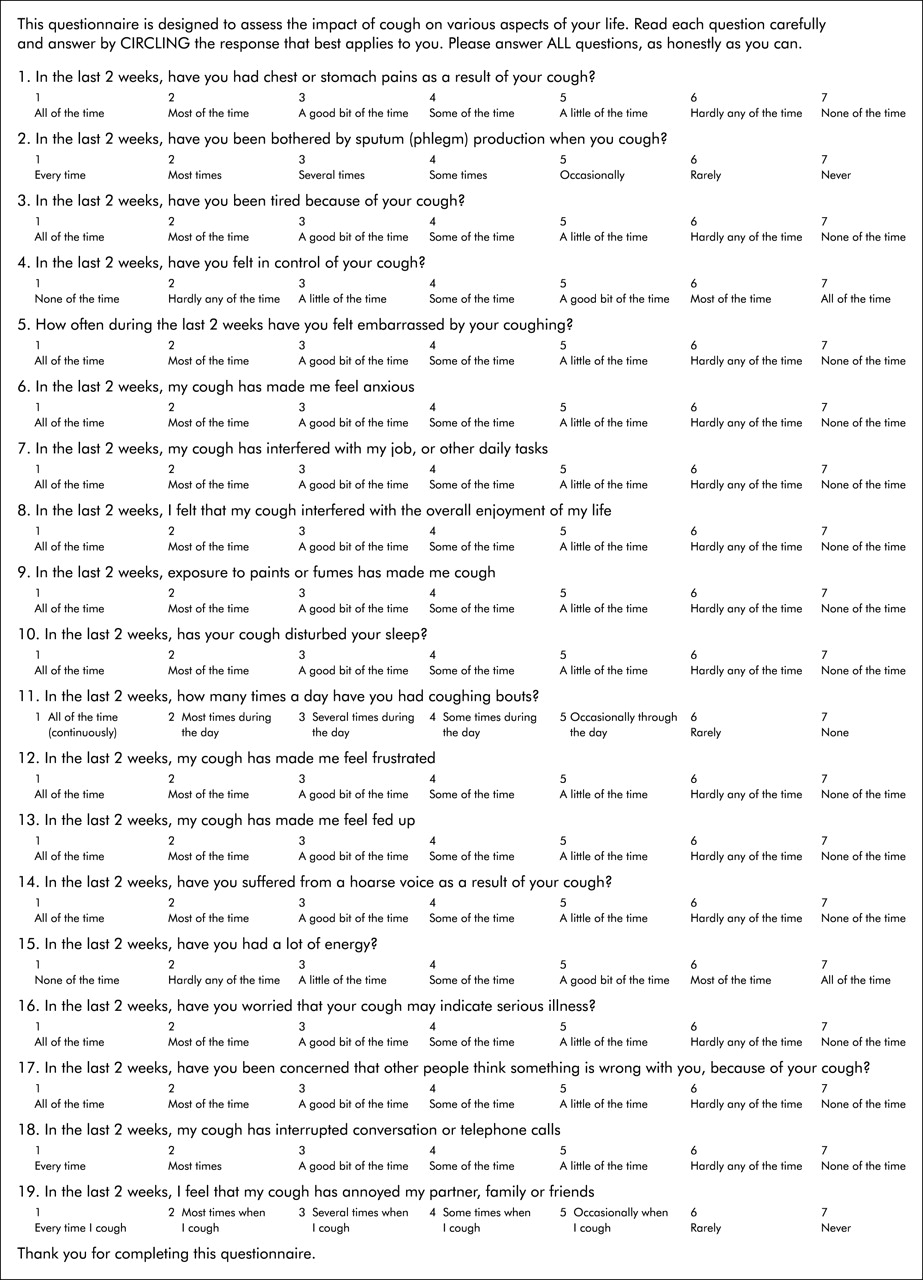


1. The GERD Symptom Assessment Scale (GSAS) is a validated symptom indicator in patients with gastroesophageal reflux disease.

11-2. Validated Evaluation Method and Analysis (Statistical Analysis Method)

The participants are randomly assigned to a high-dose PPI treatment group, a standard-dose PPI treatment group, and a placebo control group. The LCQ, Cough VAS, and GSAS are used prior to the treatment, and at 4 and 8 weeks after treatment to assess the treatment’s efficacy (only pre-treatment and at 8 weeks for GSAS).

We will compare the outcome between the groups according to the treatment dosage and duration.

Subgroup analysis will be used to compare the effects of PPI treatment in patients with typical GERD and those with non-acid (weakly acidic) reflux disease.

Appropriate descriptive statistics will be presented for each patient group, including the demographic data. Continuous variables will be expressed as means ± standard deviations, and analysis of variance (ANOVA) will be used for comparative analysis. The comparative analysis of discontinuous variables will use the chi-square test and Fisher’s exact probability method. Odds ratios will be used when the confidence interval is set to 95%. Statistical significance will be considered at a p-value of the continuous variable less than 0.05. Multivariate logistic regression analysis will be performed with items that have a p-value of 0.05 or less on univariate logistic regression analysis and basic clinical variables such as age and sex. For statistical analysis, SPSS 18.0 program will be used

**12. Criteria for Safety EvaluationーSide Effects, Evaluation Approach, and Reporting Methods**

12-1. Clinical safety before, during, and after treatment should be verified. Periodic assessments of adverse events and disease-related symptoms are made through questionnaires/surveys.

12-2. Adverse Events

The efficacy data will be analyzed according to the randomly assigned drug rather than the actual drug administered to the participant. These adverse events refer to undesirable and unintended signs (e.g., abnormal laboratory tests), symptoms, or illnesses that occur in the participants after receiving a drug used in a clinical trial. Adverse events do not necessarily need to have a causal relationship with the used drug.

12-3. Adverse Drug Reaction

An Adverse Drug Reaction (ADR) is an all-harmful and unintended reaction that occurs at any dose of a drug used in a clinical trial and cannot exclude a causal relationship with the drug used in the clinical trial.

12-4. Serious AE/Serious ADR

“Serious AE/ADR" refers to any of the following adverse reactions that occur at any dose of a drug used in the clinical trial.

- Death or life-threatening cases

- Hospital admission or prolongation of hospital stay

- Persistent or meaningful functional impairment/decline

- Associated congenital malformation or abnormalities

- Other medically significant events

12-5. Report of Unexpected Serious Abnormal Drug Reactions (SUSARs)

The investigator should promptly report any major and unexpected abnormal drug reactions (SUSAR) that occur during the course of this trial to relevant investigators, the Clinical Trial Review Board, and the Food and Drug Administration. In addition, the investigator should report additional safety information related to the report periodically until the abnormal drug reaction is completely resolved (such as the disappearance of the abnormal drug response or the inability to follow up).

1. death or life-threatening cases: Within 7 days from the date when the investigator responsible for the trial receives or reports this fact. However, in this case, additional information should be reported within 8 days from the initial reporting date.
2. All other major and unexpected abnormal drug reactions: Within 15 days from the day when the investigator responsible for the trial receives or reports this fact.

12-6. Measures to Ensure the Participants’ Safety

The institution conducting this clinical trial should have the facilities and professional staff required for the trial, and should ensure the safety of the participants in order to conduct the trial properly as specified in the research plan. The investigator should be fully aware of the adverse reactions and precautions described in this research plan. If serious adverse reactions occur during the study, the clinical trial of the participant should be discontinued as appropriate and reported to the clinical trial review committee. If side effects associated with the clinical trial are suspected, appropriate treatment compensation will be made because all the participants enrolled in the study will be covered by Samsung Fire Insurance for victim’s compensation.

**13. Research Execution Plan (Schedule)**

| Schedule | | Performance comparison | |
| --- | --- | --- | --- |
|  |  | References, research plan | |
|  |  | Study enrollment | Enrollment begins |
|  |  |  |  |
|  |  |  | Enrollment ends |
|  |  | Statistical analysis and interpretation |  |
|  |  |  | Result analysis and manuscript writing |

**14. Confidentiality of Research Materials and Monitoring Plan**

This test will be conducted in accordance with the Clinical Trial Management Standard for Medicine (GCP) and the recently revised Helsinki Declaration.

14-1. Institutional Review Board/Ethics Committee

The study protocol and any amendments thereto shall be submitted to an independent clinical trial review committee that is properly configured for formal approval of the trial implementation, in accordance with national regulations. The decision of the clinical trial review committee on the conduction of the trial shall be communicated to the investigator before the start of the trial. The investigator shall maintain a list of the clinical trial review committee members and their affiliations. Moreover, the investigator will agree with the clinical trial review committee to report any serious adverse events, life-threatening events, or deaths that occur during the trial period. The investigator should inform the clinical trial review committee if the trial is withdrawn.

14-2. Change of the Clinical Trial Plan

A formal research plan revision is needed for any major administrative changes such as the purpose and design of the study, number of study participants, and changes in test procedures. Formal revision is also necessary for any changes that could affect the patient’s safety or influence the possible benefits of the clinical trial. These changes are subject to the consent of the study population and are reviewed for approval by the clinical trials committee before implementation. Minor modifications that do not affect the clinical trial will require administrative changes. These administrative changes will be documented and they may be reported to the clinical trial review committee at the discretion of the investigator.

14-3. Patient Identification

The initials and date of birth of all selected patients will be recorded in the patient log in chronological order based on the arrival time at the first visit. If the patient is excluded from the clinical trial, the reason for exclusion is also recorded in the patient log. Each patient is assigned a random number upon enrollment. The initials and assigned number of each patient are entered in the patient case record.

14-4. Recording of Data and Maintenance

In this clinical trial, a document type of case record will be used, and all data will be entered into this document. The case record will be kept for at least 5 years after the end of the test.

**15. References**

1. Irwin RS, Curley FJ, French CL. Chronic cough: The spectrum and frequency of causes, key components of the diagnostic evaluation, and outcome of specific therapy. Am Rev Respir Dis 1990; 141: 640-647.

2. Irwin RS, Boulet LP, Cloutier MM, Fuller R, Gold PM, Hoffstein V, Ing AJ, McCool FD, O'Byrne P, Poe RH, Prakash UB, Pratter MR, Rubin BK. Managing cough as a defense mechanism and as a symptom. A consensus panel report of the Americal College of Chest Physicians. Chest 1998; 111: 133S-81S.

3. Pratter MR, Bartter T, Akers S, DuBois J. An algorithmic approach to chronic cough. Ann Intern Med 1993; 119: 977-983.

4. Jaspersen D, Kulig M, Labenz J, et al. Prevalence of extra-oesophageal manifestations in gastro-oesophageal reflux disease: an analysis based on the ProGERD Study. Aliment Pharmacol Ther 2003; 17: 1515–20.

5. Poe RH, Kallay MC. Chronic cough and gastroesophageal reflux disease: experience with specific therapy for diagnosis and treatment. Chest 2003; 123: 679–84.

6. Smyrnios NA, Irwin RS, Curley FJ, French CL. From a prospective study of chronic cough: diagnostic and therapeutic aspects in older adults. Arch Intern Med 1998; 158: 1222–8.

7. Irwin RS. Chronic cough due to gastroesophageal reflux disease: ACCP evidence-based clinical practice guidelines. Chest 2006;129(1, Suppl): 80S–94S.

8. Ours TM, Kavuru MS, Schilz RJ, Richter JE. A prospective evaluation of esophageal testing and a double-blind, randomized study of omeprazole in a diagnostic and therapeutic algorithm for chronic cough. Am J Gastroenterol 1999; 94: 3131–8.

9. Kiljander TO, Salomaa ER, Hietanen EK, Terho EO. Chronic cough and gastroesophageal reflux: a double-blind placebo-controlled study with omeprazole. Eur Respir J 2000; 16: 633–8.

10. Chang AB, Lasserson TJ, Gaffney J, Connor FL, Garske LA. Gastro-oesophageal reflux treatment for prolonged non-specific cough in children and adults. Cochrane Database Syst Rev 2006; 4: CD00-4823.

11. Chang AB, Lasserson TJ, Kiljander TO, Connor FL, Gaffney JT, Garske LA. Systematic review and meta-analysis of randomised controlled trials of gastrooesophageal reflux interventions for chronic cough associated with gastrooesophageal reflux. BMJ 2006; 332: 11–7.

12. Kiljander TO. The role of proton pump inhibitors in the management of gastroesophageal reflux disease-related asthma and chronic cough. Am J Med 2003; 115(Suppl. 3A): 65S–71S.

13. Lee JH, Cho YK, Jeon SW, Kim JH, Kim NY, Lee JS, Bak YT and The Korean Society of Neurogastroenterology and Motility. Guidelines for the Treatment of Gastroesophageal Reflux Disease. Korean J Gastroenterol 2011; 57: 57-66.

14. Dean BB, Gano AD Jr, Knight K, Ofman JJ, Fass R. Effectiveness of proton pump inhibitors in nonerosive reflux disease. Clin Gastroenterol Hepatol 2004; 2: 656-664.

15. Khan M, Santana J, Donnellan C, Preston C, Moayyedi P. Medical treatments in the short term management of reflux oesophagitis. Cochrane Database Syst Rev 2007;(2):CD003244.

16. Birring SS, Prudon B, Carr AJ, Singh SJ, Morgan MD, Pavord ID. Development of a symptom specifi c health status measure for patients with chronic cough: Leicester Cough Questionnaire (LCQ). Thorax 2003; 58: 339–43.

17. Brightling CE, Monterio W, Green RH, et al. Induced sputum and other outcome measures in chronic obstructive pulmonary disease: safety and repeatability. Respir Med 2001; 95: 999–1002.

18. Shaheen NJ, Crockett SD, Bright SD, et al. Randomised clinical trial: high-dose acid suppression for chronic cough - a double-blind, placebo-controlled study. Aliment Pharmacol Ther. 2011; 33: 225-34.

19. Birring SS. Controversies in the evaluation and management of chronic cough. Am J Respir Crit Care Med. 2011; 183: 708-15.
